# When the earth and sky dance: seismic shakes meet weather patterns

**DOI:** 10.1007/s00161-026-01485-1

**Published:** 2026-06-10

**Authors:** Alessio Kandiah, Alexander B. Movchan, Vladimir Frid

**Affiliations:** 1 Department of Civil, Environmental and Mechanical Engineering, University of Trento, Via Mesiano, 77 I-38123 Trento, Italy; 2 Department of Mathematical Sciences, University of Liverpool, Liverpool, L69 7ZL UK; 3 Sami Shamoon College of Engineering, Jabotinsky 84, 77245 Ashdod, Israel

**Keywords:** Spectral Analysis, Atmospheric Pressure Dynamics, Earth-Atmosphere Coupling, Storm Formation

## Abstract

A new modelling approach is presented, suggesting how the Earth’s hidden vibrations may be associated with global weather dynamics and atmospheric pressure variations, emphasizing the potential impact of the planet’s own beat on the formation of high-pressure patterns. The atmospheric rotational patterns of the mean sea level pressure, in connection to the development of powerful storms, are shown to be consistent with Earth’s rotational elastic dynamics and earthquake-induced oscillations. These seismic excitations are discussed in relation to storm formation and the global atmospheric patterns of high-pressure regions. The correspondence identified here is qualitative and visual; full quantitative validation through spectral analysis of pressure fields and coupled Earth—atmosphere modelling is essential future work.

## Introduction

The years 2024-2025 were marked by a series of extreme seismic events and unusually high variations of mean sea level pressure (from as low as 921 hPa to as high as 1060 hPa) which was reminiscent of the years 1883-1884, with one of the largest eruptions of Krakatoa in 1883, and the lowest ever recorded mean sea level pressure in the British Isles of 926 hPa in January of 1884, as described in the 1884 and 1930 Nature articles [1, 2]. Also, the two more recently published papers, Burt [3] and Burt [4], include the accurate account of extreme sea level readings in the British Isles over the period extending back to the 19^th^ century. These papers illustrate that extreme variations, when the mean sea level pressure drops below 960hPa or increases above 1030hPa are extremely rare, and although the accuracy of recordings and the level of detail are impressive, there is no evidence of periodic patterns in time reported in these papers. The standard frontal system, that governs the British weather, is linked to the formation of the so-called weather fronts over the Atlantic Ocean, represented by a low-pressure region and a “wedge”, whose leading side is referred to as a “warm front”, and the rear side as a “cold front”; the interior of the wedge is known as a “warm sector”. However, another type of mechanism, linked to formation of high pressure regions, instead of the low pressure, is shown to become essential in the recent years.

Recent research by Barnfield et al. [5], Kandiah et al. [6] and Kandiah et al. [7] reveals that our planet undergoes elastic oscillations [5], resonating like an immense gyroscope. The natural vibrations of the Earth ripple through the tectonic plate boundaries and echo in the atmosphere as suggested by Carbone et al. [8], revealing potential connections between Earth’s elastic dynamics and changing weather patterns. However, current observational data are insufficient to directly confirm these links, underscoring the need for targeted empirical validation through spectral analysis and atmospheric measurements to substantiate the proposed hypothesis.

**Fig. 1:**
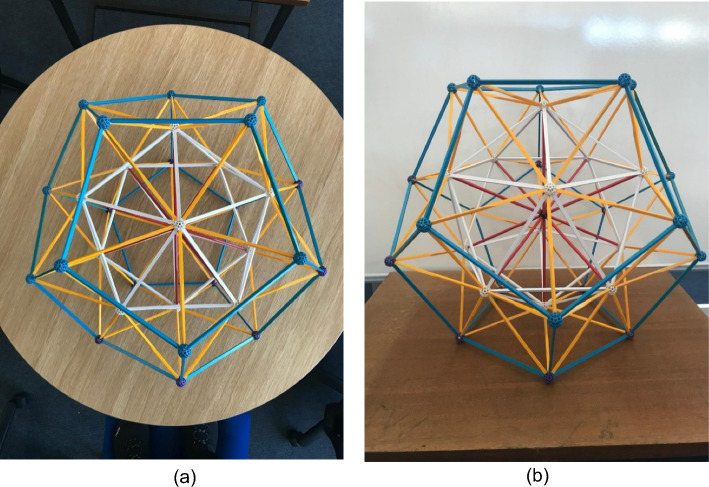
The illustrative gyroscopic icosahedron-dodecahedron lattice systems, that have been used in Barnfield et al. [5] in modelling the earthquake systems on Earth; **a** top view and **b** side view

The shallow-water wave model was used recently by Constantin and Johnson [9] to construct the hypotrochoidal patterns in the atmosphere, via the spectral analysis. The recent discrete lattice model was developed in Barnfield et al. [5] for the gyroscopic icosahedron-dodecahedron lattice system, as shown in Fig. 1, to model formation of the global vibration patterns due to earthquakes. The three-dimensional structure, shown here, includes a centred icosahedron inside the lattice and a dodecahedron as the exterior lattice structure. Such a lattice system is shown to be consistent with the global vibration patterns induced by major earthquakes.

**Fig. 2:**
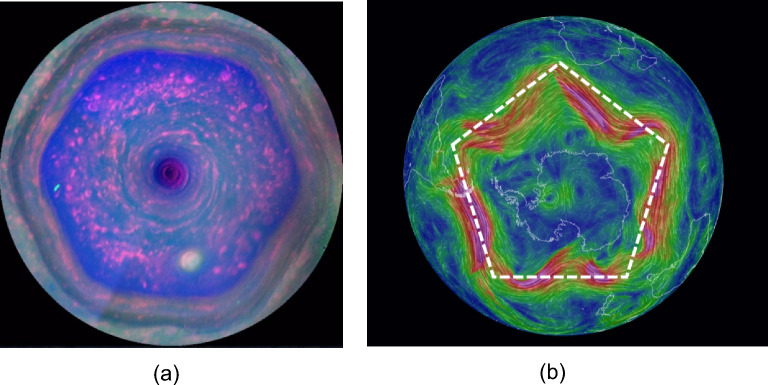
**a** Six-sided jet stream at Saturn’s North Pole from NASA’s Cassini mission. Image courtesy of NASA as of December 2025, and **b** approximate pentagonal jet stream at the South Pole of Earth on 3^rd^ January 2023 (produced using the dataset Earth_pentagon)

A striking characteristic of planetary atmospheres in the polar regions is the polygonal-like structures of fast-moving air high in the atmosphere, shaped by the combined effects of the planet’s rotation and gravity. As shown in Fig. 2a, Saturn’s North Pole hosts a persistent hexagonal jet stream, first detected by Voyager and later confirmed by Cassini NASA probes. A similar phenomenon appears on Earth in the form of an approximate pentagonal jet stream pattern at the South Pole as illustrated in Fig. 2b. These polygonal formations highlight how physical mechanisms, such as gyroscopic effects and gravitational forces, can produce highly ordered atmospheric structures.

Quantitative modelling of the coupling between ground-level vibrations and atmospheric pressure dynamics has been advanced by Godin et al. [10], who apply WKB asymptotic and full-wave numerical methods to derive resonance conditions for acoustic-gravity waves excited by seismic, oceanic, and ice-shelf sources. They demonstrate that ground-level excitation couples most efficiently into the atmosphere at buoyancy resonance frequencies in the 1–3 mHz band, and provide observational evidence of discrete bands of high correlation between ocean infragravity wave spectra and thermospheric wave activity. At the level of the elastic source, the eigenmode framework of Sakuraba et al. [11]—developed for a fluid sphere embedded in an elastic medium—establishes that resonating elastic bodies support eigenmodes of specific angular order with distinct spatial symmetries, providing the theoretical precedent for the polyhedral patterns investigated here. The present study contributes the geometric dimension absent from both prior frameworks: identifying which spatial symmetries of the rotating Earth’s elastic eigenmodes may be imprinted on the global atmospheric pressure field. A new perspective is presented here, following the suggestion by Carbone et al. [8] that atmospheric patterns may also be influenced by the Earth’s elastic vibrations. By examining mean sea-level pressure variations during recent extreme events, the analysis reveals how Earth’s hidden vibrational modes may couple with the atmosphere to shape large-scale weather dynamics.

### How earth’s rotation shapes its vibrations

It is known that the Earth vibrates with natural oscillations governed by elasticity, fluid dynamics and gravity, as described by Suda et al. [12] and Alterman et al. [13]. For a non-rotating planet, these vibrations settle into the familiar toroidal and spheroidal modes. But once rotation is taken into account, the Coriolis effect transforms the picture: the vibration modes engage a coupled oscillatory system with striking new patterns that would not exist on a non-rotating planet. These rotational effects shift the eigenfrequencies and change eigenmodes as discussed by Montagner and Roult [14], leading to phenomena such as gyroscopic frequency splitting and the Chandler wobble. The study by Barnfield et al. [5] explores different classes of vibrations in rotating elastic bodies, with physical scales that approximate Earth itself. The results revealed approximately polyhedral vibration modes in a spinning Earth-like ball, uncovering hidden resonances driven by the planet’s spin.

In the following analysis, we assume small linear elastic deformations and isotropy with uniform material properties of the ball. Under these conditions, we consider a spectral problem for the time-harmonic displacement field ${\boldsymbol{U}}\left( {\boldsymbol{x}} \right)$, where ${\boldsymbol{x}}$ represents the position vector of an elastic element, which satisfies the linearised equation

1 $$- \rho \omega^{2} \boldsymbol{U}\left( \boldsymbol{x} \right) + 2i\rho \omega \boldsymbol{\Omega} \times \boldsymbol{U}\left( \boldsymbol{x} \right) = \mu \nabla^{2} \boldsymbol{U}\left( \boldsymbol{x} \right) + \left( {\lambda + \mu } \right)\nabla \left( {\nabla \cdot \boldsymbol{U}\left( \boldsymbol{x} \right)} \right),$$

where $\rho$ is the density, $\omega$ is the radian frequency and ${{\boldsymbol{\Omega}}}$ is the angular velocity vector of the rotating body. The Lamé parameters are given by

2 $$\mu = \frac{E}{{2\left( {1 + \nu } \right)}}, \lambda = \frac{E\nu }{{\left( {1 + \nu } \right)\left( {1 - 2\nu } \right)}} ,$$

with $E$ being the Young modulus and $\nu$ the Poisson ratio. The effect of rotation appears through the Coriolis term $2i\rho \omega {{\boldsymbol{\Omega}}} \times {\boldsymbol{U}}\left( {\boldsymbol{x}} \right)$, which acts as a gyroscopic coupling. We take ${{\boldsymbol{\Omega}}} = \left( {0,0,{\Omega}_{E} } \right)^{T} ,$ where ${\Omega}_{E}$ is the Earth’s angular speed about its polar axis. The homogeneous traction boundary conditions are set on the spherical boundary of the elastic rotating ball. The material properties of the rotating elastic ball used in the numerical computations are as follows: Young's modulus $E = 110$ GPa, Poisson's ratio $\nu = 0.3$, the density $\rho = 5515$ kg/m^3^ and the angular speed ${\Omega}_{E} = 7.2921 \times 10^{ - 5}$ rad/s. These are Earth-like values consistent with PREM (Dziewonski and Anderson, [15]), and the radius of the ball is $R = 6371 \times 10^{3}$ m, corresponding to the mean radius of the Earth.

**Fig. 3:**
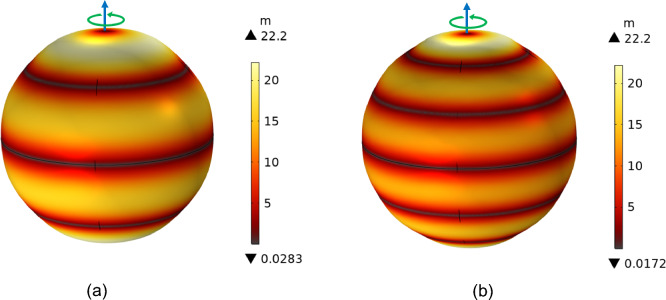
Axially symmetric eigenmodes of a rotating elastic ball, showing low-amplitude displacement bands parallel to the equatorial region for two vibration frequencies: **a** $f = 3.5188 \times 10^{ - 4}$ Hz, and **b** $f = 5.1216 \times 10^{ - 4}$ Hz. The material properties are [5]: Young’s modulus $110$ GPa, Poisson’s ratio $0.3$ and mass density $5515$ kg/m^3^. The ball rotates about its vertical axis, passing through the centre and poles, with an angular speed of $7.2921 \times 10^{ - 5}$ rad/s

At low frequencies, the rotating elastic ball reveals distinct bands that run parallel to the equator, as discussed in studies by Barnfield et al. [5], Kandiah et al. [6] and Kandiah et al. [7]. These patterns emerge since rotation induces anisotropy through the Coriolis effect, structuring the displacement field of the planet. The bands mark regions of relatively low-amplitude elastic motions, separated by boundaries where oscillations intensify. The results of the numerical computations are shown in Fig. 3 for two different vibration frequencies. On Earth, comparable banded structures appear in atmospheric circulation patterns, known as the Hadley, Ferrel and Polar cells that organise global weather systems into repeating latitudinal patterns.

**Fig. 4:**
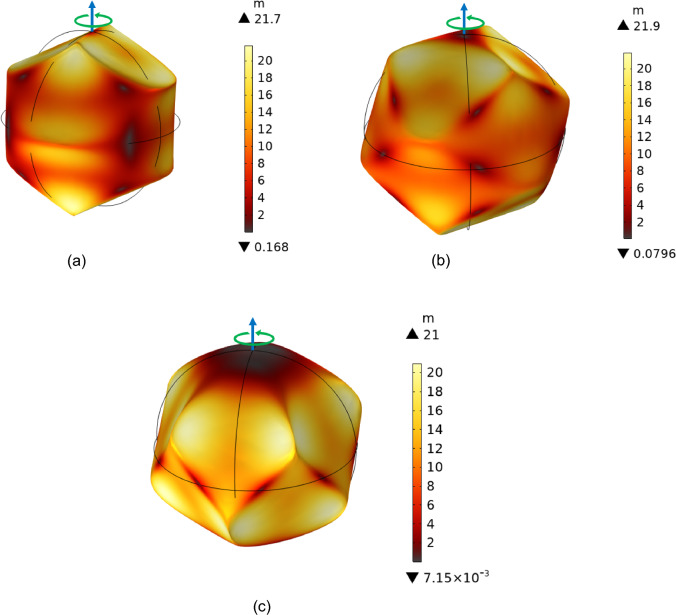
Polyhedral vibration modes of a rotating isotropic elastic ball, with rotational symmetry about the vertical $z$-axis. The material properties and the size of the ball are identical to those in Fig. 3. **a** Eigenmode with two-fold rotational symmetry for the oscillation frequency $f = 7.8146 \times 10^{ - 4}$ Hz, **b** eigenmode with three-fold rotational symmetry for the vibration frequency $f = 7.8420 \times 10^{ - 4}$ Hz, and **c** eigenmode with five-fold rotational symmetry for the oscillation frequency $f = 7.8483 \times 10^{ - 4}$ Hz

At certain frequencies, the rotating elastic ball’s vibrations may also resemble icosahedral or dodecahedral structures illustrated in Fig. 4, with pentagonal or triangular patterns across the poles and equatorial regions. These polyhedral eigenmodes depend on both the rotational speed and material properties of the rotating body.

The eigenfrequencies and eigenmodes illustrated in Figs. 3 and 4 were computed using a finite-element formulation of the linearised equations of elastic motion for a rotating isotropic elastic ball, given by (1). The Coriolis term enters via a rotating reference frame, modifying the standard toroidal and spheroidal mode classification of the non-rotating case. The displacement field is expanded in spherical harmonics for the angular structure and in radial basis functions for the radial structure, following the spectral framework of Sakuraba et al. [11], extended to a rotating elastic body with traction free boundary conditions at $r = R$. Computations were performed using COMSOL Multiphysics with the mesh refinement toward the surface and poles.

### Earth’s vibrations echoing in the atmosphere: recent global storms and pressure variations

Between October 2024 and November 2025, clusters of seismic activity across the globe occurred alongside extraordinary atmospheric disturbances and variations in pressure levels. The sequence of recent events (including typhoon Ragasa, storms Éowyn, Amy, Floris and Benjamin, and the resulting five-fold rotational patterns of high Mean Sea Level Pressure) suggests how Earth’s rotational dynamics, elastic vibrations and atmospheric pressure gradients may combine to produce large-scale weather systems. We emphasize that the connections described below are qualitative and observational; on the other hand, they are consistent with the results of the spectral analysis used to analyse non-axisymmetric vibrations of the rotating planet.

**Fig. 5:**
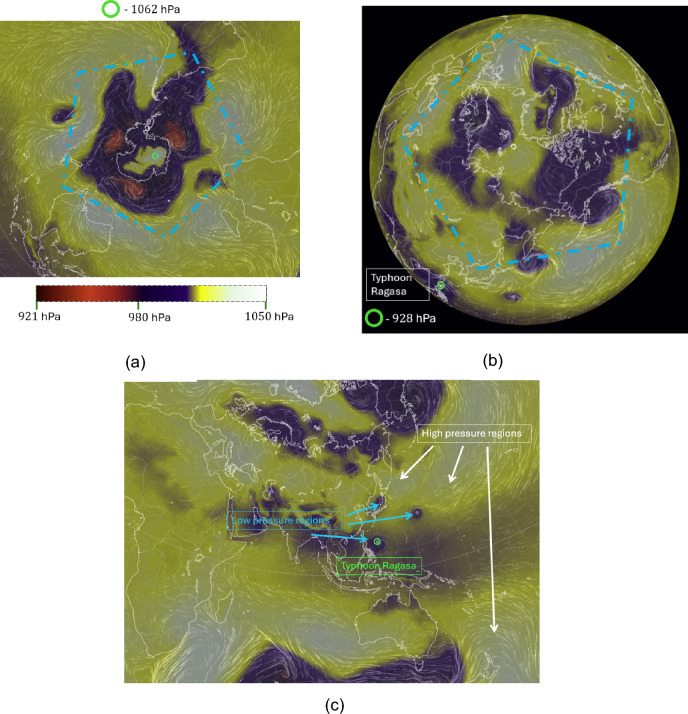
Mean sea level pressure and surface wind across the Earth’s surface prior and during Typhoon Ragasa. **a**, **b** Five-fold rotational patterns of high pressure are shown at the South and North Poles, produced using the datasets Earth_S and Earth_N. **c** Formation of low pressure vortices (including Ragasa) induced by the interaction between the high pressure systems on 20^th^ September 2025, produced from the dataset Ragasa_20

*Typhoon Ragasa* (17–25 September 2025) hit the Philippines, Taiwan and China with maximum recorded winds reaching 270 km/h and extreme pressure variations. There was no advance warning. However, there was a series of significant seismic events during 17–19 September 2025, with the strongest earthquake of Mag 7.8 in Kamchatka on 18 September 2025. According to the meteorological data, shown in Fig. 5, an approximate five-fold rotational pattern of high-pressure regions was formed in the atmosphere. On 22^nd^ September 2025, the recorded mean sea level pressure was 928 hPa in the vicinity of the Philippines as shown in Fig. 5b, which also shows the approximate pentagonal high-pressure structure. In particular, Fig. 5c shows that the vortex signifying Ragasa was formed as a result of the interaction between two closely located high-pressure systems. We note that the polygonal patterns are identified by considering the large-scale structure of the mean sea level pressure field. Within these patterns, pronounced pressure variations are apparent, allowing for the identification of an approximate centre of the pressure system.

**Fig. 6:**
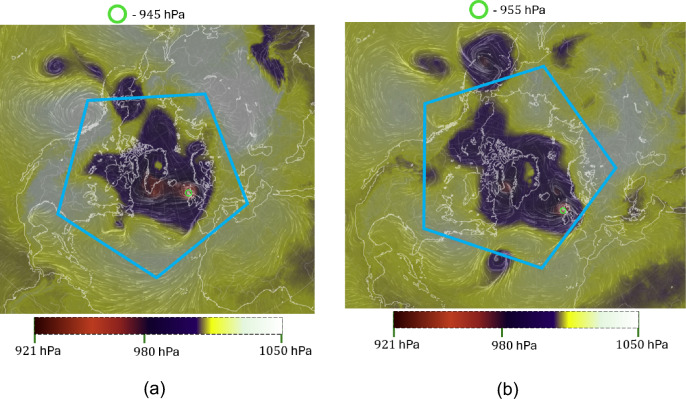
Stereographic projections of Earth’s surface winds and mean sea level pressures during (**a**) Storm Éowyn (produced from dataset Eowyn) and (**b**) Storm Amy (produced from dataset Amy). The examples also highlight the approximate five-fold symmetry patterns of the localised high-pressure systems, influenced by the vibrations of the planet and changing pressure gradients

*Storm Éowyn* (21–27 January 2025) swept across Ireland and the United Kingdom, driven by extreme pressure gradients. On 24^th^ January 2025, the mean sea level pressure varied significantly between 942 hPa and 1060 hPa within the region shown in Fig. 6a. These variations were accompanied by approximately pentagonal patterns with localised high-pressure regions in the atmosphere. Bearing in mind that extreme pressure changes were the dominant characteristics of the Storm Éowyn, there were a cluster of earthquakes prior to this storm, which included a Mag 7.1 earthquake in Tibet, China on 7^th^ January 2025, a Mag 6.8 earthquake in Kyushu, Japan on 13^th^ January 2025 and a Mag 6.0 earthquake in Yujing, Taiwan on 20^th^ January 2025. Interestingly, similar climatic conditions were observed in January 1884, when strong continuous seismic activity preceded record-low pressures of 926.5 hPa in Scotland [1, 2]. It is also worthwhile noting that in the first week of February 2025 (few days after Storm Éowyn), the Mean Sea Level pressure in the UK increased to the unusually high level of 1045 hPa (the highest Mean Sea Level Pressure recorded in the UK was 1054 hPa in 1902).

*Storm Amy* (1–6 October 2025) tracked northeast across the UK, causing severe disruption and displaying an approximately five-fold rotational pattern of high-pressure regions as shown in Fig. 6b. The following earthquakes preceded Storm Amy: Mag 7.4 and Mag 7.8 earthquakes in Kamchatka, Russia on 13^th^ and 18^th^ September 2025, respectively, Mag 6.2 and Mag 6.3 earthquakes in Zulia, Venezuela on 24^th^ and 25^th^ September 2025, respectively, and a Mag 6.9 earthquake in Leyte, Philippines on 30^th^ September 2025.

**Fig. 7:**
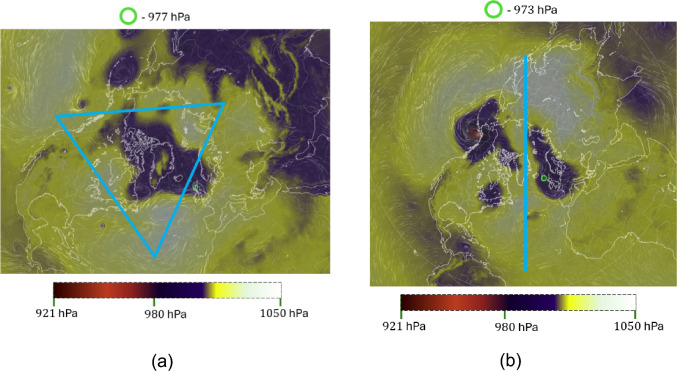
Stereographic projections illustrating Earth’s surface winds and mean sea level pressures during (**a**) Storm Floris (produced from dataset Floris) and (**b**) Storm Benjamin (produced from dataset Benjamin). The examples also demonstrate the symmetry of the localised high-pressure regions in connection with the elastic vibrations of the Earth

Remarkably, *Storm Floris* (2–4 August 2025) and *Storm Benjamin* (22–23 October 2025) were also associated with localised high-pressure systems as shown in Fig. 7, which were preceded by strong seismic activity. Storm Floris brought strong winds and heavy rain in northern Scotland, with an approximate three-fold pattern of high-pressure regions as illustrated in Fig. 7a. Storm Benjamin, which affected the UK, was surrounded by localised high-pressure regions with an approximately two-fold pattern, distinct from earlier storms, as shown in Fig. 7b. The high-pressure systems, discussed above, resemble the patterns produced by the vibration modes of the rotating elastic ball presented in Fig. 4. This is consistent with the observation that the Earth’s rotational dynamics and elastic vibrations, including seismic excitations described by Benioff et al. [16], may couple with atmospheric circulation (Carbone et al. [8]).

## Concluding remarks

These storms highlight how the elastic oscillations of the rotating Earth may resonate with atmospheric circulation, producing localised high pressure patterns, and vortices. Planetary vibrations combined with the gyroscopic effects, together with atmospheric pressure variations, may reveal the connection between Earth’s elastic rotational dynamics, vibroseismological processes and weather systems on a global scale.

Godin et al. [10] demonstrate that resonant acoustic-gravity wave coupling between the ground and the atmosphere operates selectively at discrete frequency bands rather than as a broad background feature, which is consistent with the episodic character of the polygonal pressure patterns described here.

Global observations of Earth’s free oscillation frequencies by Shimizu et al. [17], using records from three great earthquakes at stations spanning high and low latitudes, show excellent agreement with PREM. The polyhedral patterns described in the present study are interpreted as particular eigenstate configurations, which may be used as a part of the Fourier representation for a transient field, driven by seismic excitation. Full numerical simulations that couple the computation of rotating elastic eigenmodes with the atmospheric resonance framework of Godin et al. [10] present the possible direction for future work and would provide a quantitative bridge between the geometric eigenmodes described here and pressure-field observations.

## Data Availability

No datasets were generated or analysed during the current study.
